# The influence of crop production and socioeconomic factors on seasonal household dietary diversity in Burkina Faso

**DOI:** 10.1371/journal.pone.0195685

**Published:** 2018-05-17

**Authors:** Jérôme W. Somé, Andrew D. Jones

**Affiliations:** Department of Nutritional Sciences, School of Public Health, University of Michigan, Ann Arbor, Michigan, United States of America; University of South Alabama Mitchell Cancer Institute, UNITED STATES

## Abstract

Households in low-income settings are vulnerable to seasonal changes in dietary diversity because of fluctuations in food availability and access. We assessed seasonal differences in household dietary diversity in Burkina Faso, and determined the extent to which household socioeconomic status and crop production diversity modify changes in dietary diversity across seasons, using data from the nationally representative 2014 Burkina Faso Continuous Multisectoral Survey **(EMC**). A household dietary diversity score based on nine food groups was created from household food consumption data collected during four rounds of the 2014 EMC. Plot-level crop production data, and data on household assets and education were used to create variables on crop diversity and household socioeconomic status, respectively. Analyses included data for 10,790 households for which food consumption data were available for at least one round. Accounting for repeated measurements and controlling for the complex survey design and confounding covariates using a weighted multi-level model, household dietary diversity was significantly higher during both lean seasons periods, and higher still during the harvest season as compared to the post-harvest season (mean: post-harvest: 4.76 (_SE_ 0.04); beginning of lean: 5.13 (_SE_ 0.05); end of lean: 5.21 (_SE_ 0.05); harvest: 5.72 (_SE_ 0.04)), but was not different between the beginning and the end of lean season. Seasonal differences in household dietary diversity were greater among households with higher food expenditures, greater crop production, and greater monetary value of crops sale (*P*<0.05). Seasonal changes in household dietary diversity in Burkina Faso may reflect nutritional differences among agricultural households, and may be modified both by households’ socioeconomic status and agricultural characteristics.

## Introduction

Dietary diversity is essential for meeting dietary nutrient requirements [1, 2], and as such, is an important determinant of dietary adequacy, nutritional status and many associated health outcomes [2–8]. In low-income rural areas of Sub-Saharan Africa (SSA), where the majority of the population still relies on rain-fed, small-scale agricultural production as a principal source of livelihood[9, 10], poor diet quality remains an intractable challenge[11]. Smallholder farming households in these settings are especially vulnerable to seasonal changes in dietary diversity because of fluctuations in food availability and accessibility. Though staple grains and pulses are commonly available at affordable prices during the harvest and immediate post-harvest periods, stocks of these foods deplete during the lean season and can be scarce through to the next harvest [12]. Indeed, seasonal variations in food availability can have important nutritional consequences for both adults and children including reduction in energy and nutrients intakes [13–15], as well as weight loss and impaired growth [12, 15–17]. Nutrition-sensitive agricultural policies and programs commonly target improvement of dietary diversity as a key outcome with the aim of addressing seasonal gaps in availability of nutrient-rich foods. Yet, few studies have assessed temporal variability in dietary diversity or the household-level characteristics that may modify this variability in SSA.

Among the previous studies that have examined seasonal differences in dietary diversity, some have relied on data from only two time periods (i.e., post-harvest vs. lean season, or beginning of lean season vs. end of lean season) [13, 18–20], while others have used data from more than two time points [21–25]. The dynamics of food availability and access in rural subsistence settings are complex. There may be nutritionally meaningful, yet subtle changes in food availability from agricultural crop production during intervening periods (e.g., during the rainy and dry seasons), or because of complementary activities that occur outside the main agricultural cycle or at irregular intervals (e.g., livestock production, cultivation and harvest of trees or perennial crops, and gathering of wild foods or bushmeat). Therefore, it is important to assess temporal variability in dietary diversity across more than two periods of the yearly agricultural production cycle to better characterize seasonal influences. In addition, the identification of household crop production and socioeconomic characteristics that may mitigate seasonal variation in dietary diversity could provide guidance to improve the timing, targeting, and design of interventions aimed at improving diet diversity. In particular, the diversity and orientation of smallholder agricultural production have been shown to be associated with household dietary diversity in both cross-sectional studies [26–28], as well as longitudinal studies [22, 29]. Yet, it is unclear whether on-farm crop species richness or market-orientation of production may modify seasonal variation in dietary diversity by filling seasonal gaps in access to foods.

The objectives of this study are to (1) assess seasonal differences in household dietary diversity across the annual agricultural production cycle in Burkina Faso, and (2) determine the extent to which household wealth, food expenditures, market access, crop production diversity, total crop production, crop production orientation, and the education of heads of households modify these differences in household dietary diversity across seasons. We hypothesize that there will be differences in household dietary diversity across seasons, and in particular that dietary diversity will be higher during the harvest season as compared to other seasons. We further hypothesize that greater household wealth, greater food expenditures, better market access, higher education level of household head, greater household crop production diversity, total crop production, and market-orientation of production will buffer against seasonal fluctuations in household dietary diversity.

## Materials and methods

### Study design and setting

We used data from the Burkina Faso 2014 Continuous Multisectoral Survey (EMC) (Enquête Multisectorielle Continue), a nationally representative survey designed and conducted by the National Statistics Office of Burkina Faso that covers all 45 provinces of the country. The EMC applied a two-stage sampling technique. In the first stage, a total of 905 enumeration zones were randomly selected with a probability proportional to the number of households enumerated in each sampling zone. During the second stage, 12 households were randomly selected with an equal probability from each enumeration zone. Therefore, the total sample size was 10,860 households. From these households, 10,790 had food consumption data for at least one survey round and were included in the final sample for this study.

All households were surveyed during four different rounds in 2014. The four survey rounds correspond to the agricultural production cycle in Burkina Faso, that is: Round 1: mid-January to mid-March (post-harvest season); Round 2: end of April to end of June (beginning of lean season); Round 3: mid-July to mid-August (end of the lean season); and Round 4: mid-September to mid-December (harvest season). The country’s single rainy season typically extends from May to October with variation by year and agro-ecological zone, and is immediately followed by the harvest season [30]. The lean season is divided into two periods that reflect differences in food availability. In general, the planting of crops lasts from June to August and corresponds to the leanest period of the year. Shortages of staple foods and increased market prices for many food commodities in both rural and urban areas begin at the start of the lean season [31] and worsen toward the end of this season. At the end of the lean season, reduced availability of staple foods is mitigated by the availability of gathered foods (e.g., shea nuts, fruits and wild leafy vegetables), and the harvest of early crops (e.g., vegetables, fresh beans and corn). Irrigated crop production immediately following the end of the main harvest is rare and depends on water availability. Crops from this off-season production are harvested between January and March.

Burkina Faso is roughly divided into three distinct agro-ecological zones based on differences in the annual average distribution of rainfall: i) Sahelian (annual rainfall of < 600 mm during 3–4 months), ii) Sudan-Sahelian (600–900 mm during 4–5 months), and iii) Sudanian (> 900 mm during 5–6 months) [30]. Agricultural production, food and forage availability for human populations and animals differs in varying ways across each zone throughout the year [30, 32]. In the Sahelian zone, food crop and forage production are limited by the extent and distribution of rainfall. Households in this zone rely on market-purchased foods for a substantial proportion of their food needs[32]. Revenues from a variety of sources (e.g., sale of livestock, remittances, other cash transfers, and income-generating activities) are used to purchase these foods [33]. Food crop production diversity and yields are higher in the Sudan-Sahelian and Sudanian zones. Cash crops such as cotton and sesame are also commonly grown in these zones, as are many tree and perennial crops (e.g., mangoes, oranges, cashew nuts). Households in these two zones also rely less on market-purchased foods than in the Sahelian zone. Livestock production is very common in the Sahelian zone, though this activity is constrained by the availability of forages. In the Sudanian zone, crops residues and forages for livestock feed are more abundant and this zone serves as the principal location of transhumance by nomadic pastoralists from the Sahelian zone.

### Measurement of variables

Data on household-level consumption of 60 food items during the previous week were collected for each survey round. We grouped the food items reported as consumed during the previous 7 days into nine food groups: i) staple foods, ii) beans and peas, iii) nuts and seeds, iv) flesh foods, v) dairy products, vi) eggs, vii) green leafy vegetables, viii) other vegetables, and ix) fruits. The food groups were adapted from those used to construct the Minimum Dietary Diversity for Women (MDD-W) indicator, a metric developed to reflect the extent to which women are meeting their dietary micronutrient needs [34]. These food groups were selected given their strong contribution to the micronutrient adequacy of diets. Previous research in Malawi has demonstrated an association between a similar household-level dietary diversity indicator (i.e., nearly identical food groups included and based on 7-day food consumption data) and intakes per adult equivalent of energy, protein and multiple micronutrients [29]. We used nine food groups, rather than the ten used to create the MDD-W, because the food consumption data in the 2014 EMC were not sufficiently disaggregated to adequately calculate the “other vitamin A-rich fruits and vegetables” food group. Based on these food groups, we created a dietary diversity score ranging from 0–9. We did not consider the frequency of consumption or the amount of food consumed in the calculation of our dietary diversity score. For some descriptive analyses, we further disaggregated the flesh foods group into fish vs. other flesh foods. Data on the cost of food items consumed by the household were summed to obtain weekly household food expenditures for each survey round in CFA Francs, the local currency (1 CFA Franc = 0.00184 U.S. dollars). We also calculated expenditures specifically on fruits and vegetables.

Principal component analysis (PCA) was performed to create a household wealth index using data on household ownership of durable assets (i.e., radio, refrigerator, TV, telephone, bicycle, motorcycle, and car), and housing characteristics (wall, floor and roof materials, ratio of number of rooms per household member, and electricity) from the first round of the EMC [35]. All of the variables used in the PCA were dichotomized (except for the ratio of the number of rooms in the household to the number of household members), and standardized to obtain a mean of zero and a standard deviation of one before including them in the PCA. The score for each household on the first principal component was retained for purposes of creating the wealth score. Quintiles of the wealth score were created to categorize households. We also examined the education level of the household head, as well as socio-demographic information of children (i.e., age and gender) and their mothers (i.e., age, education level, and number of antenatal visits during child’s pregnancy), and household access to water and sanitation.

Data collected during the first survey round on the distance of households to the nearest market based on self-reported travel time by the most common means of transportation (i.e., 0–14 minutes, 15–29 minutes, 30–44 minutes, 45–59 minutes, ≥ 60 minutes) was used as a proxy indicator of household market access; the information on travel time from household to the nearest market was not collected based on specific transportation means and did not allow disaggregating this variable by different means of transportation. Data collected from the second survey round on remittances received by households during the previous 12 months and income generated from non-farm activities during the last 30 days were also assessed.

Plot level-data on the production of crops by each household during the 2014 rainy season were collected during the fourth round of the EMC and used to calculate the diversity of crop production. These data were primarily collected from the household head, but were also collected from another household member knowledgeable about the household’s crop production if the household head was not available. A count variable was calculated based on the number of distinct crop species cultivated by each household. Data on the amount of each crop harvested were summed to calculate the total quantity of crops produced by each household. Crop production orientation was determined for each household by calculating the percentage of harvested crops sold or planned to be sold by the household. Households for which this percentage exceeded 100% were omitted from analyses (n = 252). The amount of money received by the household from the sale of individual crops was summed across crops to calculate the total monetary value of crop sales for each household in CFA Francs.

### Data management and analysis

Statistical analyses were performed with SAS v.9.3 (SAS Institute Inc, Cary, North Carolina, USA). Final analyses included all households with available dietary data at any round of the survey. Descriptive statistics of household characteristics were calculated and were weighted to ensure representativeness at the national, regional and rural/urban levels using SAS procedures that account for sampling weights for categorical and continuous data (SURVEYFREQ or SURVEYMEANS procedures, respectively). Tertiles of the household dietary diversity score were created based on the distribution of data for all four survey rounds combined to classify households as having low, medium or high dietary diversity during each round of the survey. Based on the distribution of the percentage of harvested crops sold or planned to be sold by households, a cut-off of >5% was used to categorize households as having market-oriented production. Fifty-seven percent of households involved in agricultural activities were below this cut-off of 5%. Proportions of households consuming specific food groups in the previous seven days were used to describe the dietary patterns of households across the four seasons and were compared using a Chi-square test.

Spearman’s rank-order correlation was used to examine the association between household dietary diversity and household fruit and vegetable expenditures in order to assess the robustness of the dietary diversity indicator. Fruits and vegetables constitute a third of the food groups included in the dietary diversity indicator, and consumption of these foods have been shown to increase the diversity and quality of diets [28, 36]. Expenditures rather than quantities consumed of fruits and vegetables [28] were assessed because data on the quantities of consumed fruits and vegetables were not available for all survey rounds.

We calculated the mean dietary diversity score for households for each survey round and assessed the association between seasonal variation and household dietary diversity score using a weighted multi-level model (i.e., using the GLIMMIX procedure in SAS). The GLIMMIX procedure included the METHOD = QUADRATURE option to compute the weighted likelihood. We accounted for repeated measurements and for the complex sampling design of the EMC by adding a random effect for the enumeration cluster and for the household, and by including household sampling weights in the model statement via the OBSWEIGHT option. The model adjusted for the following predefined covariates: household head’s gender, age and education level; household size, travel time from household to nearest market, wealth status, food expenditures, income from remittances and off-farm activities, crop diversity, total crop production, total monetary value of crops sales, and crop production orientation; rural or urban location of the household residence; and regional fixed effects. Group-wise comparisons of mean dietary diversity between seasons were performed using the Tukey-Kramer test. In a subgroup analysis, we also assessed the seasonal differences in household dietary diversity by agroecological zone adjusting for the same covariates as for the analysis on the whole sample. We tested statistical interactions between seasonal variation and the following variables for the whole sample: i) education level of household head, ii) household wealth status, iii) food expenditures, iv) household crop diversity, v) travel time from household to nearest market, vi) total crop production, vii) total monetary value of crops sales and viii) crop production orientation. The effect modification results of household crop diversity, total crop production, total monetary value of crops sales, and orientation of crop production on household dietary diversity were not different in a sample restricted only to households involved in agricultural activities as compared to the sample of all households. Therefore, we only present results for the whole sample for all the tested variables. Previous evidence guided our selection of household characteristics for which to examine effect modification. For example, the education level of the head of household is a determinant of household- and individual-level dietary diversity [13], and may have a protective effect on dietary diversity during lean periods with low food availability [18]. Furthermore, agriculture may improve nutrition outcomes through both consumption of own production, and use of agriculture-generated income to purchase foods and non-food commodities [29, 37]. As noted earlier, the diversity of household crop production has been shown to be associated with household dietary diversity. Total crop production could also help households to maintain diverse diets across seasons through both subsistence and income pathways. Market-orientation of crop production and market access are also important determinants of household dietary diversity, and may play an important role in modifying seasonal differences in food availability [27, 29]. Stratified analyses were performed to further describe the interaction when the interaction term was significant at the 5% level. *P*-values < 0.05 were considered statistically significant.

## Results

### Characteristics of households

A total of 10,790 households were included in analyses. However, the sample size for each survey round differed given that dietary data were not available for all households for all four rounds. In total, dietary data were available for n = 9805 households for all rounds, n = 556 for three rounds only, n = 280 for only two rounds, and n = 149 for just one round.

Most households were rural (72.1%) and more than two-thirds of households (71.6%) were involved in agricultural activities. Heads of households were predominantly male (86.1%). The mean age of household head was 46.1± 15.4 years, and most heads of household lacked any formal education (75.3%). The median household size was 6 (interquartile range (IQR) 4–9). Among households involved in agricultural activities, the median amount of harvested crops (by weight) was 1,400 kg (IQR 684–3,000 kg), and the median number of crops cultivated during the 2014 rainy season was 3 (IQR 2–4). Median weekly household food expenditures during the four survey rounds were 7,550 CFA Francs (IQR 4,650–12,325), 6675 CFA Francs (IQR 4,175–10,600), 7,350 CFA Francs (IQR 4,400–11,900), and 7,075 CFA Francs (IQR 4,450–11,100) for the post-harvest season, the beginning of the lean season, the end of the lean season, and the harvest season, respectively.

Household dietary diversity was positively correlated with household fruit and vegetable expenditures, respectively, during the previous 7 days (rho ranged from 0.36 to 0.56 with all p-values < 0.0001) (S1 Table).

### Seasonal differences in household dietary patterns

**Fig 1:**
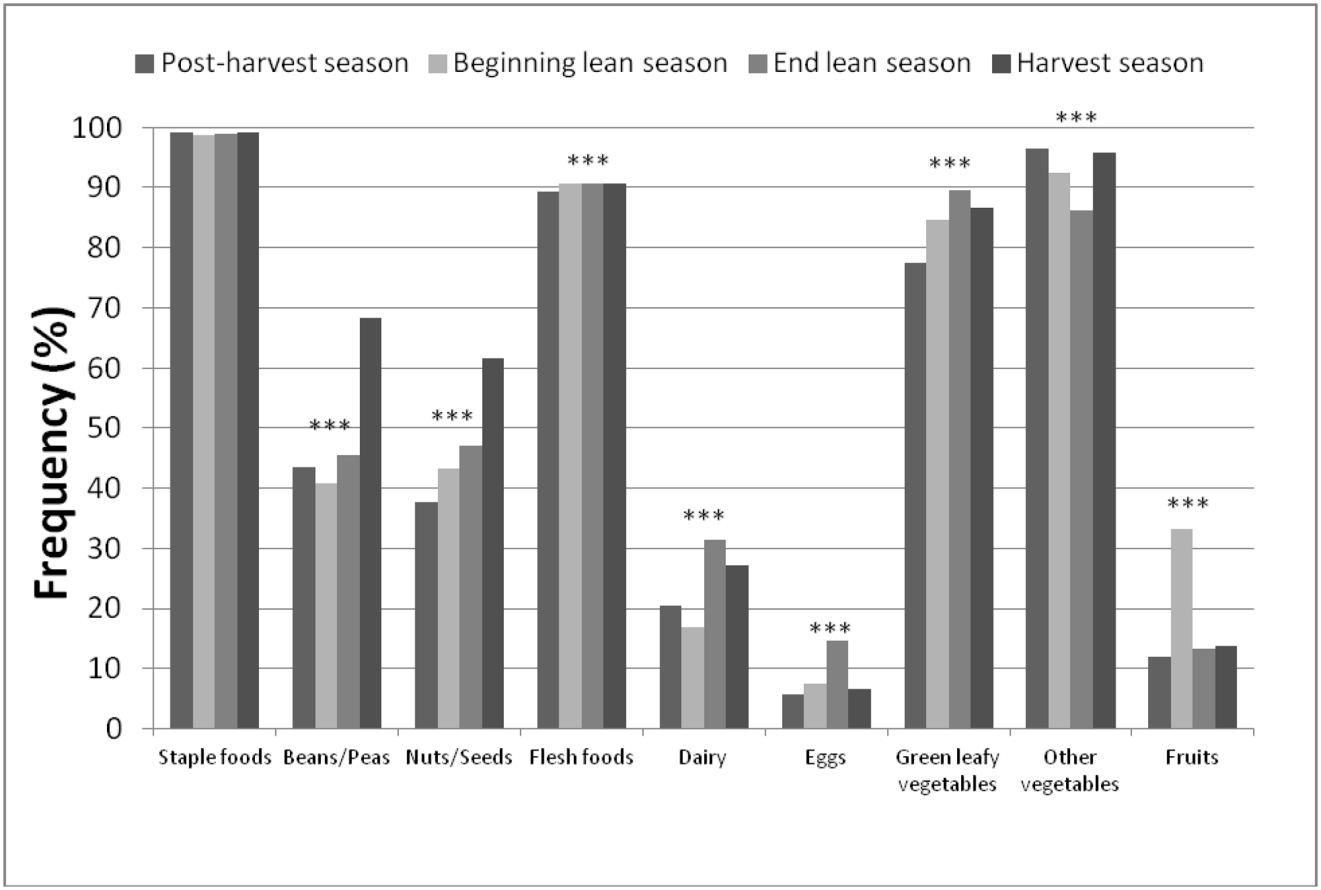
Frequency of household consumption of specific food groups across four agricultural seasons in Burkina Faso. †Values are proportions of households consuming specific food groups across the four agricultural seasons (post-harvest season: n = 10,747; beginning of lean season: n = 10,542; end of lean season: n = 10,179; and harvest season: n = 10,127). ‡P-values indicate differences among proportions across agricultural seasons within food groups from Chi-square tests: *P < 0.05, **P < 0.01, ***P < 0.001.

Staple foods (i.e., cereals, tubers and roots) were consumed by almost all households across all four seasons with no seasonal variation (Fig 1). Four-fifths or more of households regularly consumed flesh foods, green leafy vegetables, and other vegetables throughout the year. Dried fish was the predominant driver of flesh food consumption (approximately 70% of households consumed dried fish across all seasons). The consumption of green leafy vegetables was the highest during the end of lean season and that of other vegetables during the post-harvest and harvest seasons. Few households consumed dairy products, fruits, or eggs in any season (percentage range: dairy products: 16–31%; fruits: 12–32%; eggs: 6–14%). Beans and peas, and nuts and seeds were consumed by more households during the harvest season as compared to the other three seasons. The consumption of dairy products by households was highest during the end of the lean season and the harvest season, and that of fruits during the beginning of the lean season. The proportion of households consuming eggs was almost twice as high during the end of the lean season (14.1%) as compared to other seasons. Seasonal differences in household dietary patterns by agroecological zone were similar to that of the whole sample, except in the Sahelian zone where dairy products were the fourth most consumed food group across nearly all seasons. Consumption of beans and peas, nuts and seeds, and flesh foods was also lower in the Sahelian zone as compared to the Sudan-Sahelian and Sudanian zones. Furthermore, the consumption of dairy products was higher in the Sahelian zone in comparison to the other two agroecological zones.

The diets of households in the lowest tertile of dietary diversity were mainly composed of four food groups during any given season: i) staple foods, ii) green leafy vegetables, iii) other vegetables, and (iv) flesh foods (Table 1). In addition to these four food groups, more than 60% of households in the middle tertile of dietary diversity consumed beans and peas, and nuts and seeds during any season. For any given season, the proportion of households in the highest tertile of dietary diversity consuming fruits and eggs was higher in comparison to households in the two other tertiles. More than half of households in the highest tertile also consumed dairy products during any season.

**Table 1:** Proportion of households consuming foods from distinct food groups, by category of household dietary diversity and agricultural season in Burkina Faso.

Tertiles of household dietary diversity	Agricultural season											
	Post-harvest season			Beginning lean season			End of lean season			Harvest season		
	Low (n 7808)	Middle (n 1930)	High (n 1009)	Low (n 6563)	Middle (n 2380)	High (n 1599)	Low (n 6066)	Middle (n 2387)	High (n 1726)	Low (n 4773)	Middle (n 3551)	High (n 1803)
Household consumption in previous 7 days (%)												
Staple foods	98.8	100.0	100.0	98.1	100.0	100.0	98.3	100.0	100.0	98.1	100.0	100.0
Beans and peas	31.2	75.1	79.1	24.6	61.5	75.5	29.4	64.9	75.4	48.6	85.9	86.5
Nuts and seeds	20.5	78.3	91.8	22.0	68.8	92.1	24.4	73.4	89.6	30.4	85.1	97.2
Flesh foods	85.4	99.4	100.0	85.1	99.6	99.8	84.6	99.5	99.9	81.2	98.9	99.9
Dairy foods	11.0	32.1	71.4	8.1	17.3	52.0	14.5	39.8	79.7	11.3	24.0	75.1
Eggs	1.7	6.9	34.9	1.4	6.5	34.2	3.3	14.2	55.2	1.0	2.9	29.6
Green leafy vegetables	72.9	89.5	89.6	78.5	94.6	95.7	85.1	95.6	96.5	77.6	94.8	94.5
Other vegetables	95.2	99.9	100.0	88.0	99.6	99.9	77.7	97.8	99.4	91.5	99.7	99.9
Fruits	3.4	18.8	66.1	14.0	52.1	84.4	4.2	14.9	43.5	2.9	8.8	52.0

Values are proportions of households consuming one or more foods from distinct food groups in the previous 7 days, by tertiles of household dietary diversity. Tertiles of household dietary diversity were defined as the number of food groups consumed by the household in the previous 7 days: low: ≤ 5; middle: > 5 and ≤ 6: high: > 6. Chi-square tests were used to assess differences in proportions within seasons and across tertiles of household dietary diversity. Proportions were different across seasons for all food groups at the P < 0.01 level except for staple foods across seasons within the category of medium and high dietary diversity (P > 0.05), and for flesh foods across seasons within the category of high dietary diversity (P > 0.05).

### Association between seasonality and household dietary diversity

**Fig 2:**
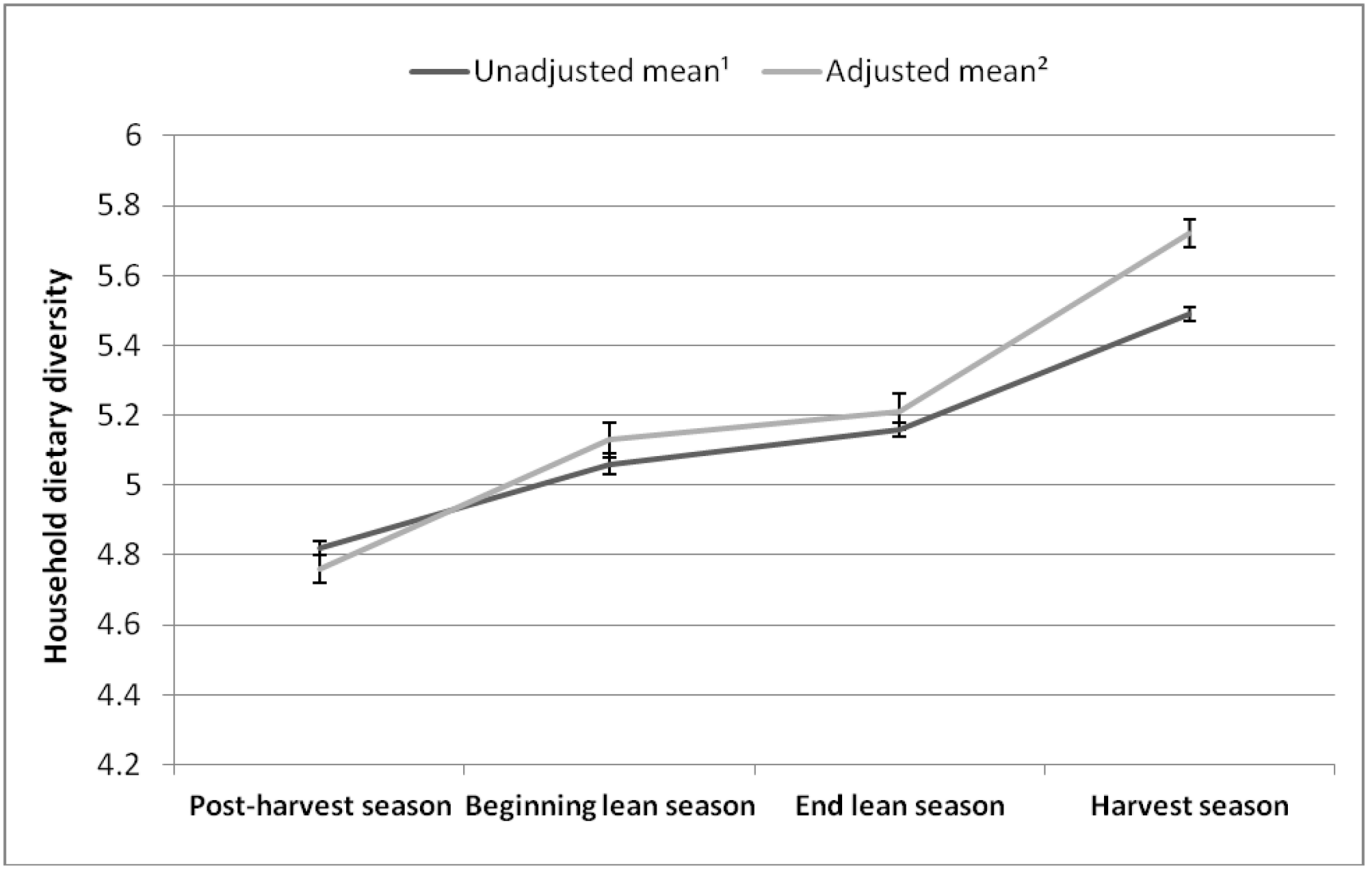
Mean household dietary diversity score across four agricultural seasons in Burkina Faso. *Values are means (standard error, SE) of household dietary diversity score during each agricultural season in Burkina Faso. †Unadjusted means are from regression models that accounted for sampling design and repeated measures of households. Adjusted means are from regression models that further adjusted for the following covariates: household head’s age, gender and education; household size, travel time from household to nearest market, total food expenditures, wealth status (quintiles), income from remittances and off-farm activities, crop diversity, total crop production, total monetary value of crop sales, and crop production orientation; region of household location and rural or urban location of household residence. ‡Unadjusted and adjusted means were different across all seasons (P < 0.001 based on Tukey-Kramer test) except for adjusted means between the beginning of the lean season and the end of the lean season (P = 0.428).

Unadjusted mean household dietary diversity score was different across the four seasons. Compared to the post-harvest season, unadjusted mean dietary diversity score was higher during both lean season periods, and higher still during the harvest season (Fig 2). After adjustment for potential confounding variables, differences in mean household dietary diversity scores among seasons remained except between the beginning of the lean season and the end of the lean season (beginning of the lean season mean score: 5.13 ± 1.46; end of the lean season mean score: 5.21 ± 1.49; *P* = 0.428, Tukey-Kramer). A similar relationship was also observed in subgroup analyses by agroecological zone; however, in the Sahelian zone differences by season in household dietary diversity were only observed between the harvest season and the three other seasons (Table 2). The proportion of households with low dietary diversity decreased from 73.6% during the post-harvest season to 49.8% during the harvest season (63.3% and 60.8% for the beginning and the end of the lean seasons, respectively). In contrast, the proportion of households with medium and high dietary diversity increased, respectively, from 17.1% and 9.4% for the post-harvest season to 34% and 16.1% during the harvest season (the proportion of households with medium dietary diversity was 22.5% and 22.7% during the beginning and the end of the lean seasons, respectively, while the proportion of households with high dietary diversity was 14.1% and 16.5% during the beginning and the end of the lean seasons, respectively) (*P* < 0.0001, χ^2^). In multiple regression analyses comparing household dietary diversity across seasons, household wealth status and food expenditures, household crop diversity, proportion of harvested crops sold or planned to be sold, and food expenditures were all positively associated with household dietary diversity (Table 3). The education level of the household head and travel time from household to nearest market were not associated with household dietary diversity.

**Table 2:** Mean household dietary diversity score across four agricultural seasons in Burkina Faso, by agroecological zone.

	Household dietary diversity								
	Postharvest season		Beginning of lean season		End of lean season		Harvest season		p-value
	Adjusted mean	SE	Adjusted mean	SE	Adjusted mean	SE	Adjusted mean	SE	
Agro-ecological zone									
Sahelian	4.80^a^	0.56	4.97^a^	0.62	5.13^a^	0.59	5.86^b^	0.62	< 0.0001
Sudan-Sahelian	4.70^a^	0.05	5.03^b^	0.07	5.09^b^	0.07	5.60^c^	0.06	< 0.0001
Sudanian	4.81^a^	0.15	5.49^b^	0.18	5.48^b^	0.16	6.02^c^	0.15	< 0.0001

Values are adjusted means and standard errors from multiple regression analyses of the association between household dietary diversity and agricultural season. Models included household head’s age and gender; household size, wealth status, total food expenditures, travel time from household to nearest market, crop diversity, total crop production, total monetary value of crop sales, crop production orientation, income from remittances and off-farm activities; and rural or urban location of household residence. Values in the same row with different superscript letters are different at the P < 0.05 level using Tukey-Kramer test.

**Table 3:** Multiple regression model results from a mixed linear model regressing household dietary diversity on seasons in Burkina Faso.

Independent variables	Household dietary diversity	SE	p-value
Seasons (post-harvest season)			
Beginning lean season	0.364	0.056	<0.0001
End lean season	0.451	0.054	<0.0001
Harvest season	0.956	0.053	<0.0001
Household head gender (male)	0.154	0.077	0.044
Household head age (years)	-0.005	0.001	0.0001
Household head education level (no formal education)			
Primary education	0.111	0.074	0.133
Secondary education	0.009	0.102	0.930
Post-secondary education	-0.139	0.157	0.379
Household size	-0.004	0.005	0.448
Crop production diversity	0.085	0.017	<0.0001
Household food expenditures (1000 FCFA)	0.024	0	<0.0001
Total crop production (kg)	3.09e-07	0	<0.0001
Income from crop sale (1000 FCFA)	5.57e-05	0	<0.0001
Household wealth status (quintile 1)			
Quintile 2	0.175	0.057	0.002
Quintile 3	0.106	0.058	0.069
Quintile 4	0.268	0.073	0.0002
Quintile 5	0.425	0.127	0.0008
Location of household residence: urban vs. rural (urban)	-0.0941	0.088	0.283
Proportion of harvest sold or planned to be sold (%)	0.002	0.001	0.030
Travel time from household to nearest market (0–14 minutes)			
15–29 minutes	0.0003	0.061	0.996
30–44 minutes	0.066	0.066	0.319
45–59 minutes	0.065	0.085	0.447
≥ 60 minutes	0.006	0.067	0.933
Income from remittance (1000 FCFA)	3.53e-05	0	<0.0001
Income from off-farm activities (1000 FCFA)	6.84e-05	0	<0.0001
Intercept	4.364	0.1733	<0.0001

Values shown are partial regression coefficients and standard errors from multiple regression analysis of the association of household dietary diversity with seasons including all variables shown as independent variables. n = 28,388. Reference categories are shown in parentheses next to independent variables.

### Household agricultural and socioeconomic characteristics modifying the association between seasonality and household dietary diversity

The education level of the household head, household food expenditures, total crop production, and total monetary value of crop sales modified the relationship between seasonality and household dietary diversity. Among nearly all levels of all four of these variables, household dietary diversity differed between the post-harvest and harvest seasons, but not between the beginning and end of the lean season (Table 4). This trend was not observed as consistently for the education level of the household head. The diversity of household diets was greater at almost all time points among households with higher food expenditures, greater monetary value of crops sale, greater total crop production, and with a household head educated at the post-secondary level. Most of these relationships showed a dose-response trend. The difference in dietary diversity between the post-harvest and harvest seasons was greater among households with household heads lacking any formal education as compared to those with a primary education (difference in adjusted mean dietary diversity between post-harvest and harvest season: no formal education: 0.99; kindergarten or primary education: 0.86). The seasonal difference in dietary diversity was also greater among households in the highest tertile vs. lowest tertile of total crop production (difference in adjusted mean dietary diversity: highest tertile: 1.1; lowest tertile: 0.88), value of crop sales (difference in adjusted mean dietary diversity: highest tertile: 1.01; lowest tertile: 0.84), and food expenditures (difference in adjusted mean dietary diversity: highest tertile: 1.07; lowest tertile: 0.97), respectively. This same finding for food expenditures was observed in subgroup analyses that included only households that remained in the same food expenditure tertile throughout the year. Household wealth status, crop diversity, crop production orientation, and travel time from household to nearest market did not modify the seasonal differences in the diversity of household diets.

**Table 4:** Results of the effect modification of education level of household head, household crop production diversity and wealth status on the seasonal differences in household dietary diversity in Burkina Faso.

Effect modifier	Household dietary diversity								
	Postharvest season		Beginning of lean season		End of lean season		Harvest season		p-value
	Adjusted mean	SE	Adjusted mean	SE	Adjusted mean	SE	Adjusted mean	SE	
Household head education									
No formal education	4.72^a^	0.04	5.11^b^	0.05	5.20^b^	0.05	5.71^c^	0.04	< 0.0001
Primary education	4.97^a^	0.23	5.34^abc^	0.28	5.37^b^	0.21	5.83^c^	0.23	< 0.0001
Secondary education	4.95^ab^	0.86	4.98^b^	0.75	5.10^ab^	0.81	5.36^a^	0.81	0.0105
Post-secondary education	4.62^a^	3.45	5.87^a^	3.46	5.70^b^	3.42	5.95^a^	3.49	< 0.0001
Total crop production*									
Tertile 1	4.62^a^	0.12	4.96^b^	0.13	4.97^b^	0.14	5.50^c^	0.15	< 0.0001
Tertile 2	4.76^a^	0.07	5.11^b^	0.07	5.07^b^	0.08	5.55^c^	0.06	< 0.0001
Tertile 3	4.83^a^	0.06	5.24^b^	0.06	5.40^b^	0.06	5.93^c^	0.05	< 0.0001
Total monetary value crops sale†									
Tertile 1	4.70^a^	0.02	5.04^b^	0.02	5.06^b^	0.02	5.54^c^	0.02	< 0.0001
Tertile 2	4.76^a^	0.07	5.27^b^	0.05	5.25^b^	0.07	5.72^c^	0.05	< 0.0001
Tertile 3	4.81^a^	0.07	5.13^b^	0.08	5.28^b^	0.08	5.82^c^	0.07	< 0.0001
Household food expenditures‡									
Tertile 1	4.26^a^	0.08	4.59^b^	0.08	4.57^b^	0.08	5.23^c^	0.07	< 0.0001
Tertile 2	4.84^a^	0.07	5.08^b^	0.08	5.11^b^	0.07	5.69^c^	0.07	< 0.0001
Tertile 3	5.16^a^	0.08	5.54^b^	0.07	5.88^c^	0.07	6.23ᵈ	0.07	< 0.0001

*Value of tertile 1 of total crop production = 808 kg; value of tertile 2 of total crop production = 2160 kg.

^†^Value of tertile 1 of total monetary value of crop sales = 13 000 FCFA; value of tertile 2 = 60 000 FCFA.

^‡^Value of tertile 1 of household food expenditure = 7400 FCFA, 6525 FCFA, 7050 FCFA, and 6975 FCFA, respectively, for the first, second, third, and fourth rounds; value of tertile 2 = 13150 FCFA, 11450 FCFA, 12825 FCFA, and 12575 FCFA, respectively, for the first, second, third, and fourth rounds. Values are adjusted means and standard errors from multiple regression analyses of the association between household dietary diversity with seasons. The main effect of each variable shown and its interaction with seasons were modeled in separate regression models. Each model also included the main effects of the other variables shown as well as household head’s age and gender; household size, wealth status, total food expenditures, travel time from household to nearest market, total crop production, total monetary value of crop sales, crop production orientation, income from remittances and off-farm activities; regional fixed effects; and rural or urban location of household residence. Values in the same row with different superscript letters are different at the P < 0.05 level using Tukey-Kramer test.

## Discussion

Our analyses showed differences in the diversity of household diets across agricultural seasons in Burkina Faso except between the beginning and the end of the lean season. Household dietary diversity was lowest during the post-harvest season, higher during both the lean season periods, and higher still during the harvest season. These seasonal differences are largely explained by the consumption of beans and peas, and nuts and seeds during the harvest season, in addition to the four food groups that are regularly consumed by almost all households year-round (i.e., staple foods, green leafy vegetables, other vegetables, and flesh foods). Rural residents in Burkina Faso commonly subsist on a thick, cereal-based porridge eaten with a sauce made of leafy vegetables and condiments such as chili, soumbala (i.e., fermented seeds of African locust bean, *Parkia biglobosa*), and dried fish [38]. As noted earlier, consumption of flesh foods was driven by consumption of dried fish. Therefore, the contribution of flesh foods to overall household dietary diversity may be overestimated given the small quantities of dried fish commonly used in the preparation of sauces for households. Subgroup analyses by agroecological zone showed similar patterns of association in the Sudan-Sahelian and Sudanian zones. In the Sahelian zone, a difference in the diversity of household diets was observed only during the harvest season as compared to the three other seasons. This observed difference could be explained by the fact that beans and peas, other vegetables, and nuts and seeds were more consumed during the harvest season as compared to the three other seasons (data not shown). In addition, dairy products were consumed to a greater extent in the Sahelian zone as compared to the other two agroecological zones, and constituted the fourth most consumed food group by households year-round in that zone. This is expected given the predominance of pastoralist livelihoods in the Sahelian zone.

Though no differences in dietary diversity between the beginning and the end of the lean season were observed, consumption of specific food groups by households did differ during these periods. More households consumed dairy products and eggs during the end of the lean season as compared to the beginning of the lean season similar to previous evidence in Burkina Faso [18]. In contrast, fruits were consumed by a higher proportion of households during the beginning of the lean season as compared to the end of the lean season. This reflects the differences in the seasonal availability of foods in Burkina Faso. Mangoes are more commonly available during the beginning of the lean season, while at the end of the lean season wild forages are more abundant with the onset of seasonal rains and therefore, milk production among cattle increases. Egg production is also highest at the end of the lean season when poultry, in extensive systems, are able to feed more intensively on foraged food. The differential contribution of dairy products, eggs and fruits to household diet diversity during the two periods of the lean season may have minimized observed differences in the household dietary diversity score across these periods. These same differences in dietary patterns during the beginning and the end of the lean season were observed in subgroup analyses by agroecological zone. In addition, the consumption of these three food groups during the lean period may explain the difference in household dietary diversity between these two seasons and the postharvest season. Another explanation may be the contribution of foraging foods (especially green leaves and wild fruits) in the diet of food-insecure households during the lean period, particularly in rural areas, as previously reported in the literature[33]; however, information collected on food items during the different survey rounds was not sufficiently disaggregated to allow assessment of this hypothesis.

We observed that lower food expenditures, and lower total crop production and monetary value of crop sales among households were associated with fewer seasonal differences in household dietary diversity between the harvest and post-harvest seasons. These findings did not align with our stated hypotheses. Households in the highest tertile of food expenditures tertile had two-fold larger household sizes as compared to households in the lowest tertile (median size: 8 (IQR: 6–12) vs. 4 (IQR: 3–6)). These households in the highest tertile of food expenditures produced the same median number of different crop species as households in the lowest tertile (3 (IQR: 2–5) vs. 3 (IQR: 2–4)), but harvested a greater amount of crops (median amount: 2,100 kg (IQR: 1,000–4,315 kg) vs. 1,000 kg (IQR: 450–2,200 kg)), and had greater income from crop sales (median income: 32,500 CFA F (IQR: 12,000–100,000 CFA F) vs. 29,750 CFA F (IQR: 7,700–99,000 CFA F)). Resource limitations among households in the lowest tertile of food expenditures likely contributed to a lower capacity to increase dietary diversity during the harvest season, and therefore the less pronounced seasonal differences in diets observed. These households with lower food expenditures also had overall lower dietary diversity than households that spent more on food. This finding confirms that of previous studies demonstrating that food expenditures, crop production, and income generated from crop sales are positively associated with dietary diversity [13, 26, 29, 37]. Furthermore, we observed a positive relationship among household wealth status [39], crop diversity [26, 28, 29, 40], crop production orientation [27] and dietary diversity of households; however, these variables did not modify seasonal differences in the diversity of household diet as hypothesized. Given the highly subsistence nature of agricultural production in Burkina Faso (i.e., 57% of households involved in agricultural activities sold less than 5% of their production), marginal increases in commercialization of household agricultural production may translate into seasonal, rather than year-round, improvements in dietary diversity because of limited infrastructure or weak institutions to support market engagement. In addition, the potential for own crop production to influence the differences in dietary diversity across agricultural seasons may have been limited due to the overall low crop diversity observed (median number of crops: 3.5 (IQR: 1–10)) and the nature of that diversity (i.e., the four most commonly produced crops were sorghum, corn, millet, and cowpea (data not shown)). So, increasing the diversity of produced crops through the inclusion of micronutrient-rich crops (vegetable crops, legumes, fruit trees) in that context could also positively impact the household dietary diversity through the pathway of own production consumption [37].

Though our study was based on a large, nationally-representative sample, and assessed dietary changes across four time points, it did have some limitations. First, due to year-to-year heterogeneity in food availability and access, our study findings, based only on data from 2014, may not be representative of longer-term variation in dietary diversity. Second, food availability during the post-harvest and lean seasons is likely to be influenced by crop production from the preceding year. We did not account for the 2013 agricultural production season in our analyses as these data were not available. Therefore, the estimated coefficients on seasonality in our regression model of household dietary diversity may be over- or under-estimated as may be the coefficients for crop production characteristics that are correlated with the previous year’s production. Third, it is also possible that crop diversity was underestimated given that data on crop production were predominantly collected from male heads of household. In some regions of Burkina Faso, women are responsible for cultivating diverse crops such as legumes and leafy vegetables, especially along the margins of cultivated fields, and near residences. These micronutrient-rich crops may have been under-reported by male household members. Finally, the household dietary diversity indicator used in this study may not reflect differences in the dietary adequacy of individuals or individual-level nutritional status. To date, only two dietary diversity indicators have been validated for use as proxies of individual-level dietary adequacy [41, 42]. In this study, higher expenditures on vegetables and fruits were consistently associated with higher household dietary diversity. Therefore, there is suggestive evidence that this score may reflect some nutritional differences. Nonetheless, even though food group diversity scores have been associated with dietary quality in previous research [1, 2, 36, 43], they have important limitations. For example, the household dietary diversity score used in this study did not differ between the beginning and the end of the lean season. Yet, there were differences in the consumption of specific food groups across these seasons. Therefore, aggregating foods into groups may mask dietary heterogeneity. In addition, the score used in this study was based on a seven-day recall. Seven-day household food consumption data have been used in previous studies to create household dietary diversity scores [26, 44]. Yet, because data from such scores are aggregated at the household level, observed dietary diversity may be quite high and the score not as discerning of variation across households as compared to individual-level dietary scores based on 24-hour recall data.

In conclusion, seasonal differences in household dietary diversity in Burkina Faso may play an important role in influencing nutritional risk among agricultural households. Development and targeting of interventions aimed at improving household dietary diversity should account for seasonal changes in household vulnerability to food and nutrition insecurity. Differences between post-harvest and harvest seasons may be particularly stark, although consumption of specific food groups (e.g., dairy foods, fruits, and eggs) shows distinct seasonal peaks during intermittent seasons. These seasonal differences in consumption patterns may be leveraged to address seasonal dietary gaps through efforts both to enhance production of specific, seasonally available foods, as well as to extend seasonal access to such micronutrient-rich foods. Improving access to education and reducing income poverty may contribute to substantially buffering families from seasonal food insecurity and related dietary deficiencies. At the same time, increasing agricultural production and farmers’ opportunities to engage in commercial production to improve their income are also important determinants of household dietary diversity. The intersections of agricultural diversification and production orientation with seasonality to influence dietary outcomes are complex, and require further research. Nonetheless, agriculture may serve as a unique lever for increasing access to diverse diets across seasons, and ensuring appropriate targeting of interventions toward the most nutritionally vulnerable populations.

## Supporting information

S1 Table Correlation between household dietary diversity and household total expenditures for fruits and vegetables during the previous 7 days for the Burkina Faso 2014 Continuous Multisectoral Survey (EMC). *DDS, household dietary diversity score; fruit expenditures, total monetary value reported by household for fruits purchase during the previous 7 days for each survey round; vegetable expenditures, total monetary value reported by household for vegetables purchase during the previous 7 days for each survey round. ‡Spearman’s rank-order correlation coefficient; total n ranging from 10,127 to 10,750 depending on variable and survey round.
(DOC)
