# Supplementary material for: The influence of crop production and socioeconomic factors on seasonal household dietary diversity in Burkina Faso
Source: PLoS One. 2018 May 17;13(5):e0195685. doi: 10.1371/journal.pone.0195685 (PMC5957435; doi:10.1371/journal.pone.0195685)
Supplement: S1 Table — *DDS, household dietary diversity score; fruit expenditures, total monetary value reported by household for fruits purchase during the previous 7 days for each survey round; vegetable expenditures, total monetary value reported by household for vegetables purchase during the previous 7 days for each survey round. ‡Spearman’s rank-order correlation coefficient; total n ranging from 10,127 to 10,750 depending on variable and survey round. (DOC) [file pone.0195685.s001.doc]

**Supporting Table 1**: Correlation between household dietary diversity and household total expenditures for fruits and vegetables during the previous 7 days for the Burkina Faso 2014 Continuous Multisectoral Survey (EMC).

|  | DDS – Fruit expenditures | | DDS – Vegetable expenditures | |
| --- | --- | --- | --- | --- |
|  | Rho‡ | p-value | rho‡ | p-value |
| First round | 0.42 | < 0.0001 | 0.36 | < 0.0001 |
| Second round | 0.56 | < 0.0001 | 0.36 | < 0.0001 |
| Third round | 0.37 | < 0.0001 | 0.46 | < 0.0001 |
| Fourth round | 0.42 | < 0.0001 | 0.37 | < 0.0001 |

*DDS, household dietary diversity score; fruit expenditures, total monetary value reported by household for fruits purchase during the previous 7 days for each survey round; vegetable expenditures, total monetary value reported by household for vegetables purchase during the previous 7 days for each survey round

‡Spearman’s rank-order correlation coefficient; total n ranging from 10,127 to 10,750 depending on variable and survey round
